# Behaviour change and physical activity interventions for physical activity engagement in community dwelling adults with chronic obstructive pulmonary disease: protocol for a systematic review

**DOI:** 10.12688/hrbopenres.13399.1

**Published:** 2021-10-01

**Authors:** Ciara Hanrahan, Julie Broderick, Terence M. O’Connor, Joseph G. McVeigh

**Affiliations:** 1 Discipline of Physiotherapy, College of Medicine and Health, University College Cork, Cork, T12 X70A, Ireland; 2 Discipline of Physiotherapy, School of Medicine, Trinity College Dublin, University of Dublin, Dublin, D08 W9RT, Ireland; 3 Department of Respiratory Medicine, Mercy University Hospital, Cork, Ireland; 4 College of Medicine and Health, University College Cork, Cork, Ireland

**Keywords:** Chronic obstructive pulmonary disease, behaviour change, physical activity, systematic review

## Abstract

**Background:** Chronic obstructive pulmonary disease (COPD) is a complex respiratory disease and the third leading cause of death worldwide. Pulmonary rehabilitation is recognised as the gold standard of care in the management of COPD, however engagement with pulmonary rehabilitation is low and maintenance of a physically active lifestyle in community dwelling adults with COPD is poor. Supporting positive behaviour change in people with COPD could help to increase their engagement with physical activity. This systematic review will examine behaviour change and physical activity interventions delivered to community dwelling adults with COPD with the aim of increasing physical activity engagement. Interventions will be mapped against Michie’s theoretical domains framework (TDF) to inform clinical practice and health policy.

**Methods: **The following databases will be searched from inception until December 2021: Web of Science, CENTRAL, MEDLINE (via EBSCO), EMBASE, APA PsychINFO, CINAHL (via EBSCO), AMED, PROSPERO, Cochrane Airways Trials Register. Reference lists of the relevant studies and grey literature will be searched using Grey Literature Report, Open Grey and Google Scholar search engines. Relevant studies will be systematically reviewed and subject to quality appraisal to determine the impact of behaviour change and physical activity interventions on outcomes of community-dwelling adults with COPD. Interventions will be mapped to Michie’s TDF and a narrative synthesis with respect to nature, effectiveness on target population and setting/environment will be provided. Findings will be reported in relation to the generalisability of the primary results and research question, and will include secondary findings on quality of life, self-reported participation in physical activity, exercise capacity, adverse events and intervention adherence. The review will be presented according to the PRISMA guidelines 2020.

**Conclusions: **This systematic review is necessary to explain the impact of behaviour change and physical activity interventions on outcomes of community dwelling people with COPD.

**PROSPERO registration:** CRD42021264965 (29.06.2021)

## Introduction

Chronic obstructive pulmonary disease (COPD) is a complex disease and the third leading cause of death worldwide ^1^. The World Health Organisation (WHO) estimates that approximately 65 million people are currently living with moderate to severe COPD globally ^2^. COPD is a heterogenous disease, defined by persistent, irreversible obstructive airflow limitation and characterised by recurrent exacerbations ^1^. The disease mainly encompasses two phenotypes; emphysema and chronic bronchitis with symptoms including chronic airways inflammation, sputum production, cough, breathlessness and reduced oxygen levels ^3^. With the exception of alpha-I anti-trypsin deficiency, COPD was thought to be caused by exposure to noxious substances, the main causative factor traditionally identified as smoking ^4^. A body of emerging evidence, however, suggests that exposure to biomass fuels and air pollution, genetic abnormalities, poor nutrition, early childhood lung infection, chronic asthma and abnormal lung development could also contribute to an accelerated decline in lung function ^4– 8^. COPD often co-exists with morbidities such as cardiovascular disease, cerebrovascular disease, diabetes and depression ^9^. People living with COPD are therefore at high risk of developing poor outcomes, such as increased healthcare utilisation and poorer quality of life (QoL) ^7^.

In COPD, patients can experience frequent exacerbations which can result in a gradual decline in physical and respiratory function over the disease trajectory ^10^. Patients can experience periodic worsening of symptoms, referred to as acute exacerbations, which are often aggravated by environmental factors and/or respiratory infections ^11^. Medical management strategies in COPD, such as the use of inhaled medications ^12– 15^, oxygen therapy ^16, 17^ and ventilatory support ^18, 19^ are commonly employed to reduce symptoms, frequency of exacerbations, hospital admissions and to improve quality of life (QoL) of those living with COPD ^20– 22^. Non-pharmacological management of COPD in the form of pulmonary rehabilitation consists of a supervised, tailored programme, often six to 10 weeks duration, offering physiological, psychological and social health benefits ^23^ and is recognised as the gold standard of care in the management of COPD ^1, 6, 24^. Programmes are based on on-going assessments of the patient’s individual disease stage, exercise capacity and co-morbidities ^25^. Pulmonary rehabilitation can be integrated into patient care at any stage of the disease, with the over-arching goal of fostering patients’ long-term engagement with physical activity and healthy behaviours ^23, 26, 27^. High attrition rates from pulmonary rehabilitation programmes are common however, resulting in lower physical activity levels among people living with COPD ^28^.

Physical inactivity leads to higher rates of morbidity, mortality and poorer QoL ^29, 30^, and people with COPD are less active than people without COPD ^31^. The majority of people with COPD reduce their levels of activity in the earliest stages of the disease, walk at a slower pace and do not generally meet the WHO recommended physical activity guidelines of 150 minutes of moderate intensity exercise per week ^29, 32– 34^. The WHO’s “Rehabilitation 2030: a call for action” outlines not only the necessity for research in the area of rehabilitation, but also for the ease of accessibility and affordability of rehabilitation for all, as essential for health management ^2^. Troosters *et al.* ^35^ report an approximate decline in steps-per-day-per-year as minus 450 in this population, the cause of this decline being multi-factorial. As such, strategies to understand and address low activity levels in this cohort must also be multifactorial.

The benefits of hospital-based pulmonary rehabilitation, for those who have the opportunity to attend, are well evidenced ^28^. These benefits, however, are not always sustained. Egan *et al.* ^36^, for example, reports that attendees with COPD (n=45) at a seven week hospital-based pulmonary rehabilitation programme experienced greatest benefits in outcomes such as breathlessness (Borg p=0.001) and physical conditioning (Incremental shuttle walk test (ISWT) p=0.013, 6MWT p=0.001) in the short and medium-term (at seven weeks and 20 weeks), but these effects were not sustained in the long-term (at 52 weeks) (Borg p= 0.011, ISWT p= 0.028, 6MWT p= 0.030). It is suggested in the literature that supporting positive behaviour change in people with COPD could help to increase engagement with physical activity ^35, 37, 38^. In order to elicit effective behaviour change, appropriate behavior change strategies must form part of physical activity interventions in those with COPD, thus facilitating translation of the benefits gained in pulmonary rehabilitation into greater life-long physical activity ^25, 35^.

Healthy behaviour change interventions can be complex, and are often targeted at a number of levels e.g. policy level, community level and/or interpersonal level in order to achieve the optimal combination for each individual person ^38^. For behaviour change interventions to succeed they should involve all stakeholders, including the patient and multidisciplinary team ^39^. The most effective behaviour change techniques are evidence-based and targeted at affecting change in the factors influencing an individual’s behaviour ^38^. While several theories exist with which to examine an individual’s behaviour, for example the theory of planned behaviour ^40^ and the transtheoretical model of behavioural change ^41^, there is a paucity of such research related to behaviour change and physical activity engagement and people with COPD.

Michie *et al.*’s ^42^ COM-B model of behaviour change is well recognised in health literature. The COM-B model sets out to contextualize an individual’s behaviour from the perspective of capability, opportunity and motivation ^43^. When used in conjunction with the theoretical domains framework (TDF) ^42^, a framework that synthesizes 33 theories of behaviour change into 14 domains related to, for example, knowledge, skills, beliefs, motivation, memory, influences and emotion ^44^, the COM-B model and the TDF combine to provide a lens through which analysis of an individual’s key behavioural determinants can take place, and thus the identification of appropriate behaviour change interventions. The COM-B and TDF have been used successfully to support behaviour change in a number of chronic conditions, for example in physical activity and counselling in obesity ^45, 46^, timely symptom presentation in cancer ^47^ and behaviour change techniques in diabetes ^48^. However, to date the behaviour change model has rarely been applied to physical activity and adults with COPD living in the community.

In one of the few studies to examine behaviours of people with COPD using the TDF, Wshah *et al.* ^37^ qualitatively explored and reported on the determinants of sedentary behaviour of 14 participants with COPD. This study found that participants lacked insight into the meaning of sedentary behaviour and, when mapped to the TDF, participants’ sedentary behaviour was mainly influenced by lack of knowledge, beliefs pertaining to capabilities, environment, resources and society. Studies in COPD have reported strategies such as tele-coaching ^49^ self-management ^50^, and counselling ^51, 52^ as effective behaviour change mechanisms to increase physical activity. In these studies, however, authors did not examine determinants of behaviour, sample sizes were small and attrition rates were high ^35, 53– 55^. It remains unclear, therefore, which are the most effective behaviour change and physical activity interventions to promote and increase engagement with physical activity in COPD. The aim of this systematic review is to evaluate behaviour change and physical activity interventions, aimed at improving outcomes for community dwelling adults with COPD. As observed in other chronic disease research, the COM-B model and TDF can work synergistically to examine the underlying determinants of behaviour and inform appropriate behaviour change strategies ^44^. Interventions from included studies will be identified and mapped against Michie’s theoretical domains framework in order to help inform clinical practice and relevant policy change for this important cohort of people with COPD.

### Objectives

1. To identify, analyse and synthesize available evidence exploring behaviour change and physical activity interventions delivered to community dwelling people with COPD.
2. To identify and map community-based behaviour change and physical activity interventions and their subsequent relationship with physical activity engagement against Michie *et al.*’s theoretical domains framework ^42^.

## Methods

This systematic review will focus on randomised controlled trials which include behaviour change and physical activity interventions for community dwelling adults with a diagnosis of COPD. This systematic review will be reported according to the Preferred Reporting Items for Systematic Reviews and Meta-Analyses (PRISMA) 2020 guidelines ^56^. The review was registered at PROSPERO (CRD42021264965) on the 29 ^th^ June 2021. This protocol is reported in line with the PRISMA-P guidelines ^61^.

### Eligibility criteria

Inclusion: Adults (18 yrs and older) with a diagnosis of stable COPD (GOLD; clinical diagnosis ^1^, best recorded post-bronchodilator ratio FEV1/FVC <0.70). Studies in English only. Where the study has mixed diagnostic groups, studies to be included if data from participants with COPD is presented separately or participants with COPD comprise >80% of mixed diagnostic groups. Randomised controlled trials that investigate any behavioural change intervention in relation to its effect on physical activity engagement or sedentary time. The intervention must consist of at least two sessions which have a physical activity focus and can be out-patient pulmonary rehabilitation, community or home-based interventions, supervised or non-supervised by a healthcare professional. Studies that do not focus on physical activity levels or sedentary time as therapeutic target will be excluded.

Exclusion: Interventions targeting caregivers, healthcare professionals or organisations. Interventions that do not include at least two behaviour change or physical activity sessions or consist of education or advice only. 

### Information sources

Searches will be carried out between 01 Sept to 01 December 2021. The following databases will be searched from inception to December 2021:

Web of Science, CENTRAL, MEDLINE (via EBSCO), EMBASE, PsychINFO, CINAHL (via EBSCO), AMED, PROSPERO, Cochrane Airways Trials Register.

Reference lists of the relevant studies and grey literature will be searched using Grey Literature Report, Open Grey and Google Scholar search engines. The search strategy will be adapted for each database.

Relevant studies will be systematically reviewed in order to evaluate behaviour change and physical activity interventions for community-dwelling adults with COPD. Interventions will be mapped to Michie’s TDF and a narrative synthesis with respect to nature, effectiveness on target population and setting/environment will be provided. Findings will be reported in relation to the generalisability of the primary results and the primary research question, and will include secondary findings on QoL, self-reported participation in physical activity, exercise capacity, adverse events and intervention adherence.

### Search strategy

Search strings will consist of free terms and controlled vocabulary. To ensure inclusion of suitable search terms and potential studies, key words from previous systematic reviews will be considered for inclusion as key words. Using a concept table, key words will be combined using the Boolean operators and / or. A sample search strategy is available as extended data ^62^.

### Study records

***Data management.*** References will be imported from Endnote Reference Manager X9 ^57^ to Covidence systematic review software 2021 for review and data extraction will be recorded in Excel.

***Selection process.*** Screening of studies initially will be based on title and abstract information and conducted by two independent investigators (CH and JMcV). Full text papers will be screened for inclusion by CH, and a 10% sample reviewed by (JMcV and JB). Where there is uncertainty regarding a papers’ suitability for inclusion, a third investigator (JB) will be involved in the process. 

The primary aim of this systematic review is to evaluate behaviour change and physical activity interventions aimed at improving outcomes for community dwelling adults with COPD. For this reason, studies that do not focus on physical activity levels or sedentary time as therapeutic target will be excluded. It is recognised by the authors that other outcome measures in the literature may be of interest to patients, clinicians and policy-makers and therefore measures including quality of life, exercise capacity, adverse events, intervention adherence and self-reported participation in physical activity will be included as secondary outcomes of the review.

Outcomes of interest will include; QoL, exercise capacity, adverse events, intervention adherence and self-reported participation in physical activity in relation to behaviour change interventions.

Where studies do not easily fit with the inclusion/exclusion criteria but where subgroup data is provided, results of the group that meet the inclusion criteria will be included. Otherwise, the study will be excluded. Study authors will be contacted where full details of interventions have not been reported or clarity is required. Studies will be excluded if details of reported interventions are not made available. 

This review will consider behaviour change and physical activity interventions for community dwelling adults with all stages of COPD and their impact, if any, on levels of, or engagement with physical activity. This study will be limited to randomised controlled trials.

***Data collection process.*** Data will be extracted from the included studies by two reviewers independently, and details entered onto a standardized abstraction form to include; publication demographics, study design, participant demographics and baseline characteristics, components and characteristics of interventions, study duration and setting, retention rates, comparator and outcomes. If relevant information from the studies is unclear or missing, published reports of the individual trials will be accessed and the individual researchers will be contacted.

***Data items.*** A template for summary of the participants, interventions, comparators, outcomes and study design for this systematic review are available as extended data ^63^, as informed by Cochrane Handbook for Systematic Reviews of Interventions version 6.2 ^58^.

### Outcomes and prioritisation

The primary outcome of the included trials will be increased physical activity engagement and/or reduction in sedentary time, when compared to usual care or no intervention. Outcome measurements may include, for example, six-minute walk test, steps per day, accelerometry, and/or self-reported changes in perception of dyspnea and functional ability. Secondary outcomes will pertain to QoL, exercise capacity, adverse events, intervention adherence and self-reported participation in physical activity.

### Risk of bias

Version 2 of the Cochrane risk of bias tool (RoB2) ^59^ for randomised controlled trials will be applied to primary outcomes to assess bias within and across studies. Studies will be assessed using five fixed domains as outlined in the RoB2, which focuses on trial design, conduct and reporting. The five domains include assessing for randomization bias, deviations from the intended interventions, missing data on the outcomes of the trial, outcome measurement bias and/or selection bias of the results reported. The RoB2 algorithm will be used to generate judgements on the study’s risk of bias as ‘low’, ‘high’, or ‘some concerns’.

### Data

***Synthesis.*** The primary interest of this systematic review is the impact of behaviour change and physical activity interventions on outcomes of community dwelling adults with COPD. The aim of the analysis will be to evaluate and characterise the reported interventions based on those deemed most effective at promoting physical activity in people with COPD. Therefore, initial analysis will include cataloguing the behaviour change interventions as reflected in the TDF ^60^.

A three-step approach will be undertaken for analysis. Initial analysis will include cataloguing the behaviour change interventions as reflected in the TDF. The behaviour change interventions will be identified and extracted from each study and summarized. Finally, changes in physical activity behaviour will be associated with relevant components of the TDF.

In order to describe the range of behaviour change interventions, summaries of the interventions with respect to nature, effectiveness on target population and setting/environment, a narrative synthesis of included studies will be provided. Findings will be reported in relation to the generalisability of the primary results and the primary research question and will also include secondary findings on quality of life, self-reported participation in physical activity, exercise capacity, adverse events and intervention adherence.

### Meta-bias(es)

A meta-analysis will be performed (using Review Manager ( RevMan) version 5.4) ^61^ if studies are homogenous in nature and a forest plot will be developed in order to summarise results. Chi-square and the I-squared statistic will be used to assess the heterogeneity of the studies to inform whether using a random effects or fixed effect model is indicated, or whether a meta-analysis is appropriate. If studies are appropriately consistent and use the same outcome measures, it may prove possible to perform a meta-analysis by pooling these results, with 95% confidence intervals and two-sided P values for every outcome. Due to the likely large range of study types, participants and outcomes that will be encountered in the literature, however, scope to produce a meta-analysis is not anticipated. Where meta-analysis is not feasible, study data will be narratively presented.

### Confidence in cumulative evidence

The GRADE working group criteria (Grading of Recommendations, Assessment, Development and Evaluations) ^62^ will be used to rate the quality of the studies identified, from very low GRADE certainty ratings to high, and reported in a table summary of findings. GRADE considerations will include limitations of studies, inconsistencies, lack of precision, indirectness and publication bias. Two investigators (CH and JMcV) will score all studies using the GRADE criteria and justify their decisions. If discrepancies exist, a third investigator (JB) will be included in the discussion to resolve the discrepancy.

## Conclusion

The primary aim of this systematic review is to evaluate behaviour change and physical activity interventions with the aim of improving outcomes for community dwelling adults with COPD. Secondary aims include identification and mapping of behaviour change interventions and their subsequent impact on physical activity levels, to Michie *et al.*’s TDF ^42^, identification of commonly used theoretical frameworks against which community-based behaviour change and physical activity interventions are mapped, and to profile the scope of interventions used for people with COPD with respect to improving outcomes.

While recent literature has identified that behaviour change and physical activity interventions may be beneficial for increasing physical activity engagement in people with COPD ^35, 55^, a systematic review of recent randomised controlled trials has yet to be published. The increasing burden of COPD on patients and healthcare systems globally warrants examination of methods to improve the QoL and management of people with COPD. This systematic review will consider behaviour change and physical activity interventions for community dwelling adults with all stages of COPD and their impact, if any, on levels of, or engagement with physical activity. It is hoped that the outcomes of this review will be applicable to patients, clinicians and policy-makers to inform their use of interventions for increasing engagement of community dwelling adults with COPD with physical activity. On completion, the results of this review will be submitted for peer-reviewed publication in this field and disseminated among relevant patient groups, clinicians and policy- makers at conferences, seminars and via social media.

## Study status

The review has not yet been initiated.

## Data availability

### Underlying data

No data are associated with this article.

### Extended data

Fishare: Sample search strategy for CINAHL.docx. https://doi.org/10.6084/m9.figshare.15173262 ^62^.

Figshare: Draft data extraction table. https://doi.org/10.6084/m9.figshare.15173265 ^63^.

### Reporting guidelines

Figshare: PRISMA-P checklist for ‘Behaviour change and physical activity interventions for physical activity engagement in community dwelling adults with chronic obstructive pulmonary disease; protocol for a systematic review’. https://doi.org/10.6084/m9.figshare.15692184. ^61^.

Data are available under the terms of the Creative Commons Attribution 4.0 International license (CC-BY 4.0).
