# Supplementary material for: Behaviour change and physical activity interventions for physical activity engagement in community dwelling adults with chronic obstructive pulmonary disease: protocol for a systematic review
Source: HRB Open Res. 2022 Feb 11;4:110. Originally published 2021 Oct 1. [Version 2] doi: 10.12688/hrbopenres.13399.2 (PMC8753571; doi:10.12688/hrbopenres.13399.2)
Supplement: Peer review history

# Peer review: Reviewer response for version 1

**DOI:** 10.21956/hrbopenres.14596.r31158

**Published:** 2022-01-11

**Authors:** Nicola Roberts

**Affiliations:** 1 Nursing and Community Health, School of Health and Life Science, Glasgow Caledonian University, Glasgow, UK

This is a very interesting proposal for a SLR and will look at the available evidence around behavior change and physical activity interventions delivered to those with COPD. The search strategy is clear.  A plan for a meta-analysis has been included, although this will be dependent on the homogeneity of the studies identified. The protocol illustrates that the search, and findings will be transparent for others to replicate. The use of the TDF framework to define the behavior change interventions and to study the changes in physical behavior will be interesting to see. The protocol reports that all stages of COPD will be considered and some indication of similarities or differences for disease severity (CAT/MRC scores)  would be good to discuss.

Is the study design appropriate for the research question?

Yes

Is the rationale for, and objectives of, the study clearly described?

Yes

Are sufficient details of the methods provided to allow replication by others?

Yes

Are the datasets clearly presented in a useable and accessible format?

Not applicable

Reviewer Expertise:

Delivery of respiratory healthcare

I confirm that I have read this submission and believe that I have an appropriate level of expertise to confirm that it is of an acceptable scientific standard.

# Response

**Published:** 2022-01-13

**Authors:** Ciara Hanrahan

**Affiliations:** University College Cork, Ireland

Thank you so much for your valuable feedback on this submission.

# Peer review: Reviewer response for version 1

**DOI:** 10.21956/hrbopenres.14596.r30705

**Published:** 2021-11-19

**Authors:** Peter Bower

**Affiliations:** 1 National Institute for Health Research (NIHR) Older People and Frailty Policy Research Unit, School of Health Sciences, Faculty of Biology, Medicine and Health, University of Manchester, Manchester, UK

Thank you for the opportunity to review this protocol, which is well written and clear. The aim is to 'examine behaviour change and physical activity interventions delivered to community dwelling adults with COPD with the aim of increasing physical activity engagement’.

 As well as a conventional review, interventions will be mapped against Michie’s theoretical domains framework in order to help inform clinical practice

 The reason for the exclusion of studies of advice, or less than 2 sessions could be justified more clearly, as that seems important in these days of ever-shorter interventions.

 What are the procedures to assess the reliability of the mapping? Is there a formal check?

 I think they intend to characterise the interventions and then make links between content and outcome. I am not clear how effective that will be if they do not assess outcomes quantitatively. They suggest meta-analysis is unlikely, but there is no requirement to POOL outcomes, even if you extract standardised effect size outcomes. I imagine assessing patterns will be very difficult if the outcomes ‘narrative’.

 I think it would help to be a little clearer on How they intend to map ‘behaviour change interventions and their subsequent impact on physical activity levels’, as the exact processes were not clear to me.

Is the study design appropriate for the research question?

Yes

Is the rationale for, and objectives of, the study clearly described?

Yes

Are sufficient details of the methods provided to allow replication by others?

Partly

Are the datasets clearly presented in a useable and accessible format?

Not applicable

Reviewer Expertise:

Health services research and self-management

I confirm that I have read this submission and believe that I have an appropriate level of expertise to confirm that it is of an acceptable scientific standard.

# Response

**Published:** 2022-01-13

**Authors:** Ciara Hanrahan

**Affiliations:** University College Cork, Ireland

Thank you so much for reviewing this submission and for your very valuable feedback. We welcome your comments and will respond as soon as possible.
